# Groundcovers and Rain Shelters Alter Co-Occurrence Patterns among Ground Beetle Communities in an Organic Raspberry Crop

**DOI:** 10.3390/insects13050413

**Published:** 2022-04-27

**Authors:** Gaétan Moreau, Charles Comeau, Jean-Pierre Privé

**Affiliations:** 1 Département de Biologie, Université de Moncton, Moncton, NB E1A 3E9, Canada; 2 Department of Agriculture, Aquaculture and Fisheries New Brunswick, 381 Killam Drive, Moncton, NB E1C 3T1, Canada; charles.comeau2@gnb.ca; 3 Plant Medic Inc., Cocagne, NB E4R 3G8, Canada; plantmedic.jp@gmail.com

**Keywords:** Carabidae, rain shelters, reflective groundcovers, *Rubus idaeus*, species co-occurrence

## Abstract

**Simple Summary:**

Rain shelters and reflective groundcovers improve the economic and environmental sustainability of organic fruit crops affected by a number of plant pathogens. In this study, we examined whether these structures also affect the communities of species that inhabit the soil surface, particularly ground beetles, in organic red raspberry crops. Our results indicate that ground beetle species richness, activity and functional traits differed in the presence of rain shelters and reflective groundcovers, but these effects were relatively minor. Thus, this study suggests that these structures, which have known benefits against plant pathogens, had no detrimental impact on ground beetle communities.

**Abstract:**

The use of rain shelters and reflective groundcovers has been shown to improve the economic and environmental sustainability of organic fruit crops prone to rain-driven epidemics of phytopathogens. Here, we tested whether these structures affect communities of epigean species. To this end, we studied rain shelters and white, synthetic reflective groundcovers placed in a red raspberry organic cropping system in New Brunswick, Canada, during two subsequent summers to assess their independent and combined effects on ground beetles (Coleoptera: Carabidae). 18,445 ground beetles belonging to 54 species were collected. Rain shelters and reflective groundcovers altered patterns of ground beetle species richness, activity density and functional diversity compared to the control, but to a limited extent. Thus, this study suggests that these structures, which have known benefits against phytopathogens, have no detrimental impact on epigean fauna.

## 1. Introduction

**Figure 1:**
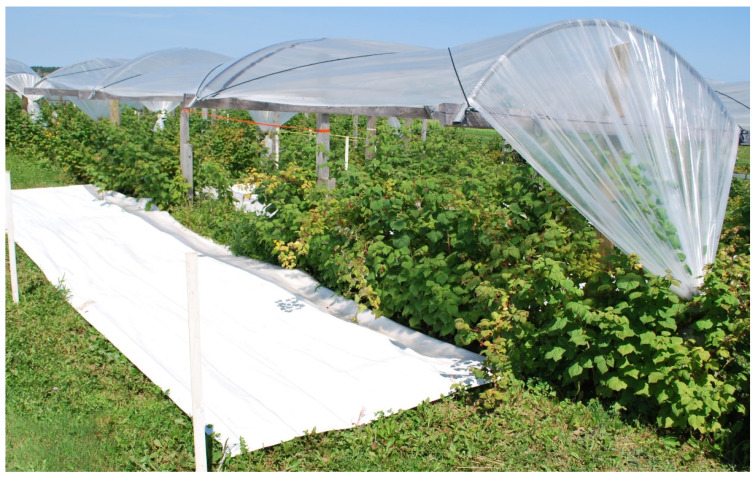
Rain shelter and reflective groundcover in a raspberry plantation.

In organic fruit crops, the control of phytopathogens often represents a considerable challenge because of the limited availability and high cost of organic fungicides. Therefore, structures covered with a polyethylene film, hereinafter called rain shelters, have been developed to prevent contact between rain water and the crop canopy in those fruit crops that are prone to rain-driven epidemics of phytopathogens [1,2]. Because rain shelters can cause a decline in photosynthesis through a possible loss of light energy reflected or absorbed by the shelter (e.g., [3]), they are commonly supplemented with synthetic reflective groundcovers to increase the amount of light captured by crop canopies [4]. Ground covers are not plastic mulches, but tarps hung a few inches above a patch of soil that will not be mowed during the growing season. The combination of both treatments, however, does artificially alter habitat structure (Figure 1).

Considering that complex habitats offer a greater variety of niches, we would expect agroecosystems making use of these structures to harbor richer and potentially more stable communities [5,6,7]. Indeed, the “habitat heterogeneity hypothesis” states that an increase in habitat heterogeneity should be associated with an increase in animal species diversity, because the number of partitionable niche dimensions expands in structurally complex habitats [6,8]. However, several studies have now indicated that an increase in the number of different habitats does not always lead to an increase in species diversity (e.g., [9,10,11]). According to Tews et al. [12], this can arise because the relationship between habitat heterogeneity and animal species diversity is affected by the definitions and measures used to describe species diversity and habitat heterogeneity. Thus, habitat heterogeneity sometimes refers to structural (i.e., physical) components, compositional components (i.e., in terms of plant species), or both [5,13]. However, structural (e.g., [14,15,16,17]) and compositional (e.g., [18,19,20]) heterogeneity do not necessarily have the same effects on ecosystem components (e.g., microclimate, niche distribution, refuges, interspecific interactions, food and host availability).

Herein, we assess whether red raspberry (*Rubus idaeus* L.; Family: Rosaceae) cropping systems managed organically with rain shelters and reflective groundcovers have altered assemblages of epigean species. Rain shelters and reflective groundcovers enhance the vertical structure of arable habitats and have the potential to create refuge microhabitats for epigean species that are preyed upon by birds and other vertebrate predators [21,22]. At the same time, their use does not result in a significant change in plant species composition in red raspberry crops (G. Moreau, personal observations), probably because groundcovers allow sufficient light, air and water to pass through to allow for sod survival (see Figure 1). To investigate the effects these structures have on epigean species, we used ground beetles (Coleoptera: Carabidae) as model organisms. Ground beetles are among the most abundant arthropods in agroecosystems and are frequently used as biological indicators in these habitats because they reflect the species richness of other insect orders [23,24,25]. They are considered an important family of beneficial insects because they serve as a food source for farmland birds and contribute to pest control [26]. We predicted that rain shelters and groundcovers will lead to an increase in ground beetle diversity and functional diversity because of an increase in the multidimensionality of the habitat. In return, we expected that higher species diversity will alter interspecific interactions among ground beetles [27].

## 2. Materials and Methods

### 2.1. Study Sites

We conducted the study during two subsequent summers in two cultivated red raspberry plantations of the variety ‘Killarney’ located in Saint-Joseph-de-Kent (46°25.97′ N, 64°46.17′ W), New Brunswick, Canada. Both plantations were surrounded by fields of medium-sized herbaceous plants, grasses, and shrubs (e.g., *Trifolium* spp., *Ranunculus* spp., *Prunella* spp., *Phleum* spp., *Plantago* spp., *Agrostis* spp.). The first plantation was established in 2007 and the second one was established in 2008, 50 m north of the first one. Both plantations consisted of five 60 m raspberry plant rows oriented North–South. The first and last rows were used as guard rows while the three interior rows were used for our experiment. The two plantations had 3-meter-wide inter-rows composed of perennial weeds, were trickle-irrigated, and were managed organically. To suppress phytopathogen epidemics while complying with organic management procedures, both plantations were treated three times a year with baking soda (3 kg/ha) in a water solution. The first plantation was used for field work in 2008 and both plantations were used in 2009.

### 2.2. Treatments

The four treatments were defined as: (1) control (i.e., no structure), (2) reflective groundcovers alone, (3) rain shelters alone, and (4) the combination of rain shelters with reflective groundcovers. Treatments including synthetic reflective groundcovers had 9-m-long, 2-meter-wide strips of Extenday^®^ (Extenday New-Zealand Ltd., Auckland, New-Zealand) type 4273 laid down between raspberry rows over the sod and secured as per the methods described in [28]. Rain shelters consisted of a wood and steel structure covered with a polyethylene film that prevented rainwater from reaching the raspberry canopy (Figure 1). The rain shelters covered 9-meter-long sections of rows and were built following the methods described in [2]. An experimental unit was defined as a 10-m section of a raspberry row and the adjacent inter-rows on each side. The experimental units located in the same rows were separated by a buffer section of at least 6 m. In both plantations, the four treatments were replicated three times and arranged in a constrained randomized design in which units treated with reflective groundcovers were placed in adjacent experimental rows to avoid the reflection of light in other experimental units that did not include reflective groundcovers (see Figure S1). In both years, treatments were set up during the last week of May and removed in early September. The allocation of treatments to experimental units was identical in both years.

### 2.3. Ground Beetle Sampling and Identification

Ground beetles were live-trapped using pitfall traps in 2008 and 2009 as per the methods described in [28]. Briefly, these traps were made from two plastic cups inserted into one another. The bottom of each cup had been replaced by a fine mesh to let the rainwater out. A plastic funnel was placed in the interior cup to prevent the escape of the captured beetles. The traps were inserted in holes dug in the ground so that the rim of the funnel was even with the soil surface (see Figure S2). A square of chicken wire was placed over the traps to dissuade vertebrate predators from feeding on trap content. Aside from an occasional spider, slug or earwig, only adult ground beetles were collected in pitfall traps during the study. Each experimental unit had two pitfall traps, one on each side of the raspberry plant row, located less than 30 cm from the center of the unit. The content of these two traps represents one pitfall trap sample. The interrow distance between pitfall traps was 3 m. The pitfall traps were emptied every three to four days from June to September during both years, for a total of 204 pitfall trap samples (51 per treatment).

Captured adult beetles were identified using [29]. The feeding guilds and average length of species were determined from the literature [21,29], except for the length of *L. pilicornis*, *S. thoracicus*, *S. pumicatus* and *S. impunctatus*, which were not available and were instead measured from specimens.

### 2.4. Statistical Analyses

To verify whether our sampling intensity was adequate to recover most of the ground beetles captured with this method of trapping, we computed rarefaction curves (Mao Tau) for the four treatments using the *vegan* package in R version 3.5.2 [30]. To examine the effects of treatments and yearly accumulated degree days over 5 °C on species richness, activity density, average beetle length and beetle function, generalized additive models were carried out using the *gam* function from the *mgcv* package in R. Given that the number of trapped ground beetles is a function of both individual activity and population density [31], we use the term “activity density” instead of abundance hereinafter. The beetle function is an index we developed to determine whether the proportion of herbivorous, omnivorous, and carnivorous species differed among treatments. The index was based on the following values: herbivore = 0, omnivore = 0.5, carnivore = 1; the sample frequency was obtained by multiplying the number of beetles in each category by the index and dividing the resulting sum by the number of beetles in the sample. A first-order autoregressive correlation structure was assumed for the effect of yearly accumulated degree days. Years and blocks were included as fixed effects. The same model performed using Julian days instead of cumulative degree-days gave similar but less explanatory results and is therefore not presented. To determine which treatment levels were different, post-hoc comparisons were performed using the *glht* function from the *multcomp* package. To produce an ordination of treatment and ground beetle data, a 2-D NMDS was carried out using the *metaMDS* function from the *mgcv* package. The *metaMDS* package applies a square root transformation to the data and uses Bray-Curtis distances (a NMDS performed using Morisita-Horn distances yielded a higher stress value and is not presented). The NMDS was performed with species collected 10 times or more. Patterns of species co-occurrence among species were compared with statistical randomizations of the species occurrence data using the software EcoSim [32]. EcoSim tests for nonrandom patterns of species co-occurrence in a presence/absence matrix. For each treatment, 5000 random matrices from the original matrices of co-occurrence were created. The C-score statistic [33], which is based on Diamond’s [27] notion of checkerboard distributions between all possible pairs of species, was estimated to identify aggregation (low C-score) or segregation (high C-score) in species co-occurrence. Only species collected in a given treatment were included in EcoSim for the analysis of this treatment. To account for the multiple tests of hypothesis in EcoSim, a correction for false discovery rate [34] was applied. Species co-occurrence was further examined using the package *cooccur* [35] to identify the species involved in interactions.

## 3. Results

**Figure 2:**
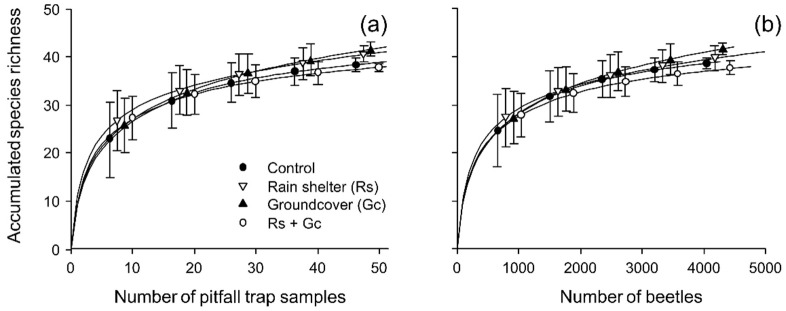
Sample-based (**a**) and rescaled sample-based (**b**) rarefaction curves (±standard deviation) for the accumulation of ground beetle species in the different treatments.

18,445 adult ground beetles belonging to 54 species were collected during this study (Table 1). The rarefaction curves suggested that our sampling intensity was sufficient to recover most of the ground beetle species that could be collected using pitfall traps because the species accumulation curves of the different treatments approached a plateau (Figure 2). Three species represented nearly 75% of all captured ground beetles, namely *Harpalus* *rufipes*, *Pterostichus melanarius,* and *Amara familiaris* (Table 1).

### 3.1. Effects of Treatments on Ground Beetles

**Figure 3:**
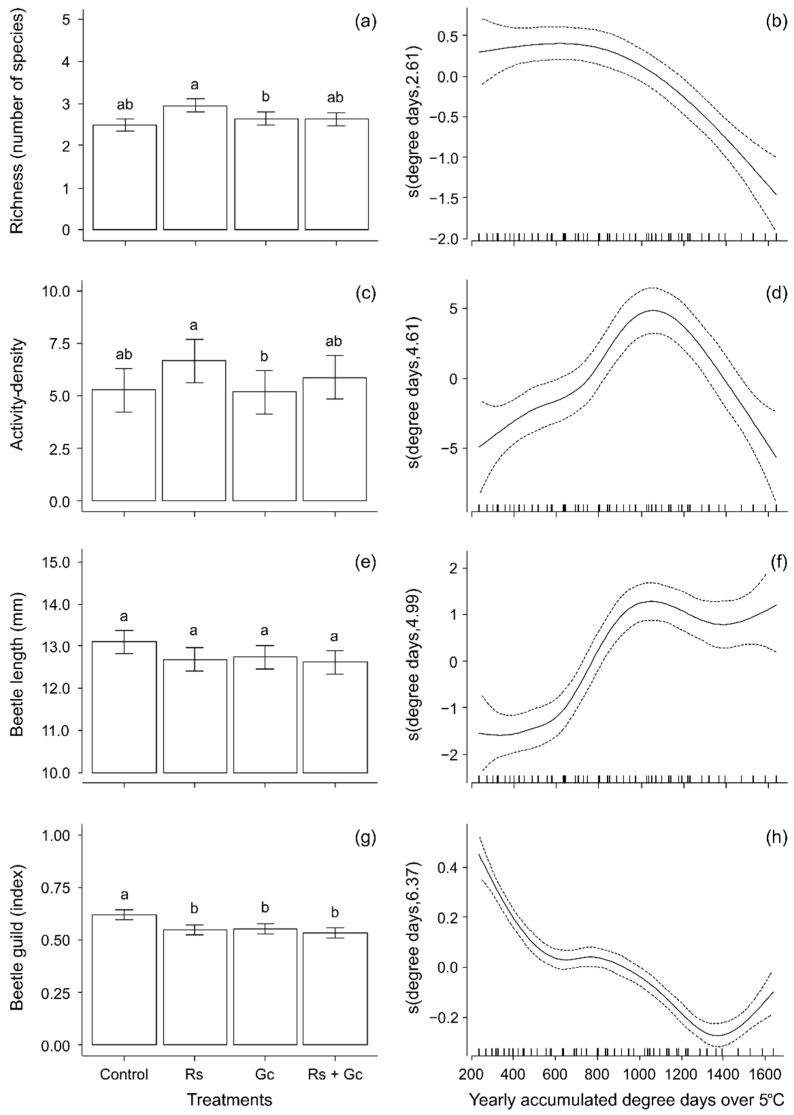
Effects of treatments and yearly accumulated degree days on ground beetle species richness. (**a**,**b**), ground beetle activity density; (**c**,**d**), ground beetle length; and (**e**,**f**) ground beetle feeding guild (**g**,**h**) per trap per sampling event. The left panel presents model predictions of treatment effects ± SEM (**a**,**c**,**e**,**g**) based on block 1 in the Year 2009 at 950 degree-days, while the right panel shows estimated smoothing curves for the models. Different letters above bars indicate statistical differences at the 0.05 level.

All measures of compositional and functional diversity used in this study fluctuated over the summer (Table 2; Figure 3b,d,f,h). An interaction between rain shelters and reflective groundcovers resulted in slightly higher species richness in areas that included a rain shelter only (Table 2; Figure 3a). Rain shelters, alone and in combination with groundcovers, also increased ground beetle activity-density (Table 2; Figure 3c). Average species length was not affected by treatments (Table 2; Figure 3e), but both rain shelters and/or groundcovers resulted in a lower incidence of predatory ground beetles (Table 2; Figure 3g). The latter effect was associated with an increased abundance of *Harpalus rufipes*, a herbivorous species, in treated plots (Table 1).

### 3.2. Effects of Treatments on Ground Beetle Occurrence, Co-Occurrence and Interactions

**Figure 4:**
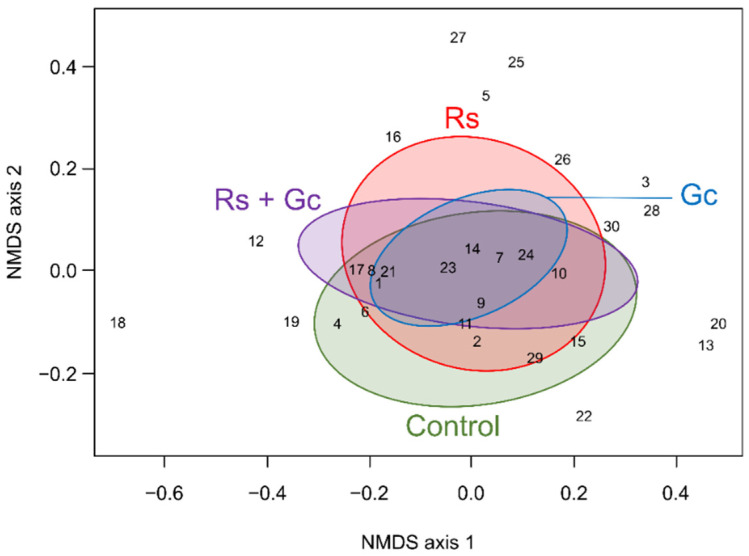
2-D non-metric multidimensional scaling (NMDS) ordination of ground beetle species abundance per treatment. Numbers correspond to the species identity in Table 1.

**Figure 5:**
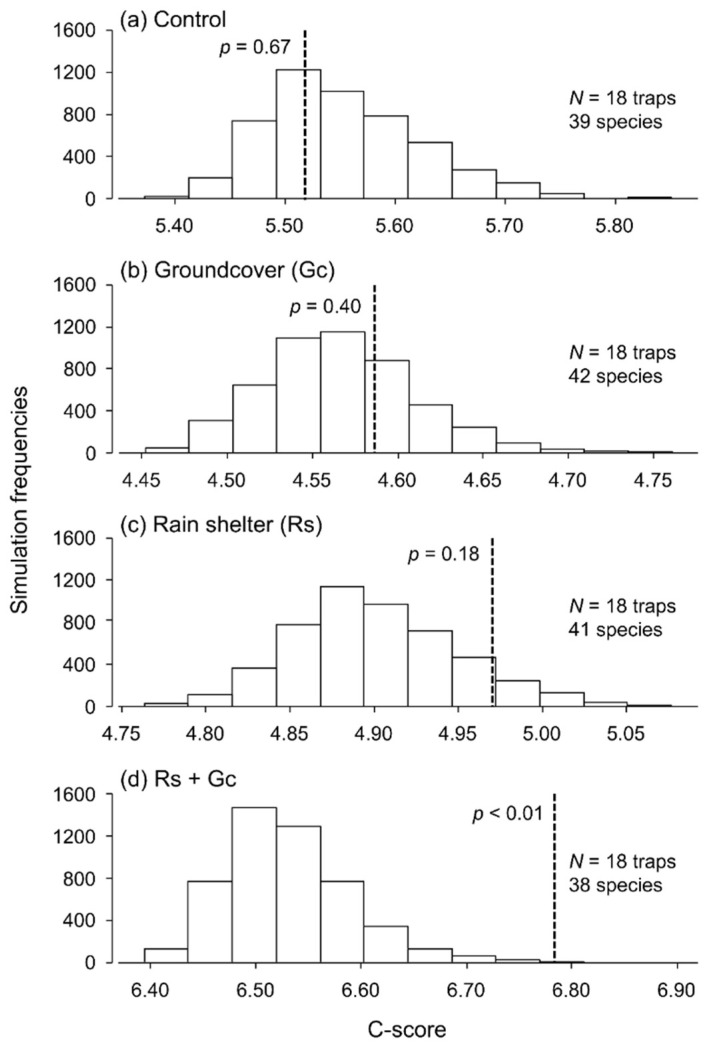
Co-occurrence patterns among the assemblages in (**a**) control areas, (**b**) areas with groundcovers, (**c**) areas with rain shelters and (**d**) areas with both groundcovers and rain shelters. The C-scores of the empirical assemblages found in the four treatments are shown by a dotted line with a corresponding *p*-value. The frequencies of the C-scores associated with null models are shown by histograms.

There was little evidence that treatments were structuring the species assemblages in a non-metric dimensional scaling (2-D NMDS; stress value = 0.072), as ellipses corresponding to different treatments stacked on top of each other (Figure 4). Figure 5 shows the C-scores of the observed communities for each of the treatments and the histograms of randomized communities. In control areas (Figure 5a), the C-scores of observed and randomized communities were similar, indicating that there was no evidence of aggregation or segregation in species co-occurrence. The introduction of structures in raspberry crops resulted in little changes when groundcovers (Figure 5b) or rain shelters (Figure 5c) were introduced alone. However, in areas where rain shelters were combined with groundcovers, significant changes in species co-occurrence were detected (Figure 5d). Analysis of ground beetle species co-occurrence showed a decline in the number of observed species pairs, a decrease in positive species interactions, and an increase in negative species interactions with higher structural complexity (see Supplementary File S1). The species causing these negative interactions were, in order of frequency, *P. melanarius* (*n* = 18 pairs), *Carabus granulatus* (*n* = 6 pairs), *H. rufipes* (*n* = 3 pairs), *A. bifrons* (*n* = 3 pairs), as well as *A. aulica*, *Anisodactylus nigrita*, *C. nemoralis*, *H. rubripes*, and *Stenolophus comma* (*n* = 1 pair each).

## 4. Discussion

Investigating the effects of rain shelters and reflective ground covers while accounting for variability associated with temporal changes in carabid community cycles allowed us to document effects not detected previously [2,28]. Areas treated with a rain shelter only caught on average of 0.5 more species per trap per sampling event than control areas, which translates approximately into a 20% increase in species richness. Then, areas treated with reflective groundcovers, alone or in combination with a rain shelter, caught on average 1.1 additional individuals per sampling event, translating into a 20% increase in activity density. Few changes in functional diversity were documented, with beetle size remaining largely the same between treatments, while herbivores were more prevalent in treated areas due to an increase in the abundance of *H. rufipes*. The latter change may be explained by the higher productivity of raspberry plants in treated areas, since *H. rufipes* readily feeds on raspberry crops in Canada [36]. Overall, these results indicate that rain shelters and reflective ground covers alter epigean communities at small spatial scales, but the changes detected were limited.

Because the NMDS suggested that none of the species exhibited a preference for a given treatment, negative associations between pairs of ground beetle species in sites of high structural complexity (i.e., Rs + Gc) indicate that competitive interactions occurred between ground beetle species in these areas. Our best interpretation of this result is that individuals likely accumulated over time in areas where structures were present, perhaps because conditions were more favorable or less unfavorable (lower predation pressure), which ultimately led to an increase in (1) the number of individuals, (2) interactions between individuals, and (3) interspecific interactions. Negative interactions (i.e., interactions that occurred less often than by chance) were caused, in most cases (i.e., 94% of the time), by medium or large exotic species introduced from Europe (i.e., *P. melanarius*, *C. granulatus*, *C. nemoralis*, *H. rufipes*, *H. rubripes*, *A. bifrons*, *A. aulica*). Several European ground beetle species are dominant in Canadian agroecosystems, a success that is largely attributed to their competitiveness and flexibility in habitat use [37]. This may be a typical indication of human disturbance, but it also indicates that the interactions between species in these communities are affected by habitat structure. The need to determine whether community structures result from species interactions or stochastic effects and habitat heterogeneity has previously been stressed in the literature [38]. The results of this study indicate that it might not be that simple to determine, as species interactions can themselves be under the influence of habitat heterogeneity.

Changes in the ground beetle complex associated with structures could indicate a change in abiotic or biotic factors that rendered the presence of these structures more favorable for ground beetles or favored the colonization/immigration of these organisms later in the summer. Indeed, the two structures might have been perceived as refuges against predation by birds, or for escaping detrimental environmental conditions that appeared around mid-summer. Other factors that can affect ground-beetle-specific microhabitat or refuge selection include food availability, microclimatic conditions, and the presence of competitors [39,40]. By restricting rainfall over a specific area, rain shelters have the potential to decrease soil moisture directly underneath them, and consequently to create patches of dryer soil within agricultural habitats, conditions that could favor certain ground beetle species. However, none of the effects detected in this study were at the species level, indicating that these processes mostly occurred at the community level.

Aspects that would warrant further study are the documented change in the species complex associated with the treatments. Thus, with increasing habitat complexity, fewer predators were recorded, while higher numbers of *H. rufipes*, a seed predator [21], were observed. Although this species is now considered naturalized, there is still a risk that it may negatively impact native carabid assemblages. Fewer predators might also result in lower pest suppression, while a higher abundance of seed predators might result in higher weed control. In addition, differences were detected between the two study blocks. Although little can be inferred other than speculations, it is possible that this is associated with the difference in age of the two plantations, or a gradient toward the river that runs north of the study area.

Another aspect to consider is that the experimental plots were of a small size, which is to be expected given that berry fields in our area are typically of a very limited size. Despite this, systematic differences were detected, and samples from traps located at the edge of the study areas produced similar results to those located in the center of study areas. This indicates that even at a small scale, it is feasible to document treatment effects in ground beetles.

Lastly, it is important to mention that this study focused on epigean fauna and it is possible that canopy fauna is affected differently since the presence of reflective groundcovers and rain shelters alters plant growth and disease incidence [2]. On this subject, the literature offers little insight, as few studies have examined the effect of rain shelters on insects, compared to the large literature on growth tunnels. To date, the few studies available have indicated that rain shelters can have positive, negative or neutral effects on canopy insects [41,42,43].

## 5. Conclusions

One of the underlying principles of organic agriculture consists of preserving species diversity within arable habitats which can, in return, render beneficial services [44,45,46]. Our results suggest that the use of reflective groundcovers and rain shelters, alone or in combination, has a limited impact on the epigean community when using ground beetles as reference organisms in an organic raspberry crop. Although a few exotic species largely dominated our ground beetle communities (i.e., *H. rufipes*, *P. melanarius* [29]), as was documented in previous field studies in the same geographic area [2,28], a high diversity of ground beetles was sampled in this agroecosystem compared to a non-organic crop field of raspberries in the same area [28]. Since reflective groundcovers and rain shelters have also been shown in previous studies to benefit crops and decrease phytopathogen pressure [2,4,47], we suggest that they represent promising tools to improve crop health and yield, without detrimental effects on epigean communities.

## Figures and Tables

**Table 1:** Species names, total number collected per treatment, feeding guild and average body length for every species during this study in New Brunswick, Canada. The feeding guilds (H = herbivorous, C = carnivorous and O = omnivorous) and average length of species were determined from the literature [21,29], except for the length of *L. pilicornis*, *S. thoracicus*, *S. pumicatus* and *S. impunctatus*, which were not available and were instead measured from specimens.

Species	Control	Ground-Cover (Gc)	Rain Shelter (Rs)	Rs + Gc	Total	Feeding Guild	Body Length (mm)
*Harpalus rufipes* (DeGeer)	2014	2369	2492	2622	9497	H	13.4
*Pterostichus melanarius* (Illiger)	761	588	500	573	2422	C	15.5
*Amara familiaris* (Duftschmid)	385	400	529	436	1750	O	6.4
*Agonum muelleri* (Herbst)	281	233	260	228	1002	C	8.4
*Amara fulva* (O.F. Müller)	69	150	145	112	476	H	9.2
*Carabus granulatus* Linnaeus	107	117	129	98	451	O	20.0
*Bembidion properans* (Stephens)	89	87	173	72	421	C	3.9
*Harpalus affinis* (Schrank)	73	72	134	134	413	O	10.3
*Carabus nemoralis* O.F. Müller	109	99	78	76	362	C	23.5
*Amara aenea* (DeGeer)	112	67	94	81	354	O	7.5
*Poecilus lucublandus* (Say)	47	50	48	33	178	O	11.5
*Amara bifrons* (Gyllenhal)	10	15	71	46	142	H	6.2
*Harpalus rubripes* (Duftschmid)	30	20	56	35	141	-	10.4
*Bembidion quadrimaculatum oppositum* Say	22	40	37	27	126	C	3.3
*Harpalus somnulentus* Dejean	26	28	28	40	122	C	9.9
*Agonum placidum* (Say)	14	10	25	7	56	O	7.8
*Loricera pilicornis* (Fabricius)	7	16	13	15	51	C	7.3
*Stenolophus comma* (Fabricius)	4	35	2	6	47	O	6.6
*Agonum cupripenne* (Say)	15	8	10	13	46	C	8.4
*Amara cupreolata* Putzeys	9	9	19	7	44	O	7.3
*Amara neoscotica* Casey	11	8	11	14	44	-	7.9
*Amara aulica* (Panzer)	7	6	21	8	42	H	12.7
*Harpalus herbivagus* Say	7	9	16	5	37	O	8.5
*Clivina fossor* (Linnaeus)	5	4	9	18	36	O	6.0
*Amara latior* (Kirby)	9	1	18	4	32	C	9.9
*Amara apricaria* (Paykull)	3	8	13	5	29	O	7.8
*Amara avida* (Say)	4	5	8	3	20	O	8.6
*Amara otiosa* Casey	2	4	7	1	14	-	7.8
*Anisodactylus nigrita* Dejean	5	4	2	3	14	C	11.8
*Elaphropus incurvus* (Say)	2	2	4	4	12	C	2.3
*Amara littoralis* Dejean	0	2	3	4	9	O	8.0
*Carabus serratus* Say	0	1	2	5	8	O	20.0
*Harpalus pensylvanicus* (DeGeer)	0	1	5	0	6	O	12.7
*Amara patruelis* Dejean	3	0	1	0	4	C	8.7
*Blemus discus* (Fabricius)	1	1	1	1	4	C	5.0
*Chlaenius sericeus* (Forster)	3	0	1	0	4	C	13.8
*Pterostichus mutus* (Say)	2	2	0	0	4	C	11.5
*Agonum octopunctatum* (Fabricius)	1	0	0	2	3	C	8.0
*Anisodactylus sanctaecrucis* (Fabricius)	1	2	0	0	3	O	9.6
*Stomis pumicatus* (Panzer)	1	1	0	1	3	-	7.5
*Dyschiriodes globulosus* (Say)	0	1	1	0	2	C	2.9
*Omophron americanum* Dejean	0	2	0	0	2	C	6.1
*Synuchus impunctatus* (Say)	0	1	1	0	2	O	9.4
*Amara pallipes* Kirby	0	1	0	0	1	H	7.1
*Amara quenseli* (Schönherr)	0	0	0	1	1	O	6.8
*Anisodactylus rusticus* (Say)	1	0	0	0	1	O	10.0
*Apristus subsulcatus* (Dejean)	0	0	1	0	1	-	3.8
*Bembidion versicolor* (LeConte)	0	1	0	0	1	C	3.2
*Chlaenius emarginatus* Say	0	0	0	1	1	O	13.6
*Notiophilus aquaticus* (Linnaeus)	0	0	1	0	1	C	5.3
*Oxypselaphus pusillus* (LeConte)	1	0	0	0	1	C	6.1
*Pterostichus adstrictus* Eschscholtz	0	0	0	1	1	C	11.3
*Stenolophus conjuctus* (Say)	0	0	1	0	1	C	3.8
*Stenolophus thoracicus* Casey	0	0	1	0	1	-	3.8

**Table 2:** *F*-values of additive models from Figure 3. In all cases, *df* = 1, except for the smoothing parameter (degree days) where *edf* = 2.61, 4.61, 4.99 and 6.37 for richness, activity-density, beetle length and beetle function, respectively.

	Block	Year	Degree Days	Treatments
Richness	64.64 **	6.30 *	24.17 **	5.97 **
Activity-density	36.23 **	49.96 **	15.87 **	2.67 *
Beetle length	39.17 **	0.07	30.09 **	2.14
Beetle function	30.49 **	84.83 **	74.56 **	8.72 **

* 0.05 > *p* > 0.01. ** *p* < 0.01.

## Data Availability

The data presented in this study are available on request from the corresponding author. Data are not publicly available due to ongoing unpublished studies.

## Supplementary Materials

The following supporting information can be downloaded at: https://www.mdpi.com/article/10.3390/insects13050413/s1, Figure S1: Study design; Figure S2: Pitfall trap design; Supplementary File S1: Pairwise interactions between species for the four treatments.

## References

[1] Xiao C.L., Chandler C.K., Price J.F., Duval J.R., Mertely J.C., Legard D.E. (2001). Comparison of epidemics of *Botrytis* fruit rot and powdery mildew of strawberry in large plastic tunnel and field production systems. Plant Dis..

[2] Comeau C., Privé J.-P., Moreau G. (2012). Beneficial impacts of the combined use of rain shelters and reflective groundcovers in an organic raspberry cropping system. Agr. Ecosyst. Environ..

[3] Rohloff J., Nestby R., Folkestad J.A., Iversen T.-H. (2004). Influence of rain cover cultivation on taste and aroma quality of strawberries (*Fragaria ananassa* Duch.). J. Food Agric. Environ..

[4] Privé J.P., Russell L., Leblanc A. (2008). Use of Extenday reflective groundcover in production of ‘Gala’ apples (*Malus domestica*) in New Brunswick, Canada: 1. Impact on canopy microclimate and leaf gas exchange. N. Z. J. Crop Hortic. Sci..

[5] Bazzaz F.A. (1975). Plant species diversity in old-field successional ecosystems in Southern Illinois. Ecology.

[6] Rosenzweig M.L. (1995). Species Diversity in Space and Time.

[7] McCann K.S. (2000). The diversity–stability debate. Nature.

[8] MacArthur R.H., Wilson E.O. (1967). The Theory of Island Biogeography.

[9] Ralph C.J. (1985). Habitat association patterns of forest and steppe birds of northern Patagonia, Argentina. Condor.

[10] Sullivan T.P., Sullivan D.S. (2001). Influence of variable retention harvests on forest ecosystems. II. Diversity and population dynamics of small mammals. J. Appl. Ecol..

[11] Reynolds H.L., Mittelbach G.G., Darcy-Hall T.L., Houseman G.R., Gross K.L. (2007). No effect of varying soil resource heterogeneity on plant species richness in a low fertility grassland. J. Ecol..

[12] Tews J., Brose U., Grimm V., Tielbörger K., Wichmann M.C., Schwager M., Jeltsch F. (2004). Animal species diversity driven by habitat heterogeneity/diversity: The importance of keystone structures. J. Biogeogr..

[13] MacArthur R.H., MacArthur J.W. (1961). On bird species diversity. Ecology.

[14] Huffaker C. (1958). Experimental studies on predation: Dispersion factors and predator-prey oscillations. Hilgardia.

[15] Moreau G., Eveleigh E.S., Lucarotti C.J., Quiring D.T. (2006). Ecosystem alteration modifies the relative strengths of bottom-up and top-down forces in a herbivore population. J. Anim. Ecol..

[16] Moreau G., Eveleigh E.S., Lucarotti C.J., Quiring D.T. (2006). Stage- specific responses to ecosystem alteration in an eruptive herbivorous insect. J. Appl. Ecol..

[17] Mourant A., Lecomte N., Moreau G. (2018). Indirect effects of an ecosystem engineer: How the Canadian beaver can drive the reproduction of saproxylic beetles. J. Zool..

[18] Pimentel D. (1961). Species diversity and insect population outbreaks. Ann. Entomol. Soc. Am..

[19] Siemann E., Tilman D., Haarstad J., Ritchie M. (1998). Experimental tests of the dependence of arthropod diversity on plant diversity. Am. Nat..

[20] Goguen J., Moreau G. (2015). Exogenous and endogenous factors acting on the spatial distribution of a chrysomelid in extensively managed blueberry fields. Agr. For. Entomol..

[21] Larochelle A., Larivière M.-C. (2003). Natural History of the Ground-Beetles (Coleoptera: Carabidae) of America North of Mexico.

[22] Holland J.M., Thomas C.F.G., Birkett T., Southway S. (2007). Spatio-temporal distribution and emergence of beetles in arable fields in relation to soil moisture. Bull. Entomol. Res..

[23] Ings T.C., Hartley S.E. (1999). The effect of habitat structure on carabid communities during the regeneration of a native Scottish forest. For. Ecol. Manag..

[24] Rainio J., Niemelä J. (2003). Ground beetles (Coleoptera: Carabidae) as bioindicators. Biodivers. Conserv..

[25] Borchard F., Buchholz S., Helbing F., Fartmann T. (2014). Carabid beetles and spiders as bioindicators for the evaluation of montane heathland restoration on former spruce forests. Biol. Conserv..

[26] Holland J.M., Thomas C.F.G., Birkett T., Southway S., Oaten H. (2005). Farm-scale spatiotemporal dynamics of predatory beetles in arable crops. J. Appl. Ecol..

[27] Diamond J.M., Cody M.L., Diamond J.M. (1975). Assembly of species communities. Ecology and Evolution of Communities.

[28] Comeau C., Privé J.-P., Moreau G. (2013). Effects of reflective groundcovers on ground beetles (Coleoptera: Carabidae) in red raspberry (*Rubus idaeus*) cropping systems. J. Appl. Entomol..

[29] Bousquet Y. (2010). Illustrated Identification Guide to Adults and Larvae of Northeastern North American Ground Beetles (Coleoptera: Carabidae).

[30] R Core Team (2018). R: A Language and Environment for Statistical Computing.

[31] Tretzel E. (1955). Technik und Bedeutung des Fallenfanges für ökologische Untersuchungen. Zool. Anz..

[32] Gotelli N.J., Entsminger G.L. (2001). EcoSim: Null Models Software for Ecology. Version 7.0.

[33] Stone L., Roberts A. (1990). The checkerboard score and species distributions. Oecologia.

[34] Hochberg Y., Benjamini Y. (1990). More powerful procedures for multiple significance testing. Statist. Med..

[35] Griffith D.M., Veech J.A., Marsh C.J. (2016). Cooccur: Probabilistic species co-occurrence analysis in R. J. Stat. Softw..

[36] Campbell J.M., Sarazin M.J., Lyons D.B. (1989). Canadian Beetles (Coleoptera) Injurious to Crops, Ornamentals, Stored Products, and Buildings.

[37] Holland J.M., Holland J.M. (2002). Carabid beetles: Their ecology, survival and use in agroecosystems. The Agroecology of Carabid Beetles.

[38] Farnon Ellwood M.D., Manica A., Foster W.A. (2009). Stochastic and deterministic processes jointly structure tropical arthropod communities. Ecol. Lett..

[39] Lövei G.L., Sunderland K.D. (1996). Ecology and behaviour of ground beetles (Coleoptera: Carabidae). Annu. Rev. Entomol..

[40] Kromp B. (1999). Carabid beetles in sustainable agriculture: A review on pest control efficacy, cultivation impacts and enhancement. Agr. Ecosyst. Environ..

[41] Masters G.J., Brown V.K., Clarke I.P., Whittaker J.B., Hollier J.A. (1998). Direct and indirect effects of climate change on insect herbivores: Auchenorrhyncha (Homoptera). Ecol. Entomol..

[42] Kratky B.A. Plastic-covered Rainshelters for Vegetable Production in The Tropics. Proceedings of the 33th National Agricultural Plastics Congress of the American Society for Plasticulture.

[43] Lim K.H., Gu M., Song J.H., Cho Y.S., Kim W.S., Kim B.S., Jung S.K., Choi H.S. (2014). Growth, fruit production, and disease occurrence of rain-sheltered Asian pear trees. Sci. Hortic..

[44] Pimentel D., Hepperly P., Hanson J., Douds D., Seidel R. (2005). Environmental, energetic, and economic comparisons of organic and conventional farming systems. Bioscience.

[45] Tscharntke T., Klein A.M., Kruess A., Steffan-Dewenter I., Thies C. (2005). Landscape perspectives on agricultural intensification and biodiversity–ecosystem service management. Ecol. Lett..

[46] Holzschuh A., Steffan-Dewenter I., Tscharntke T. (2008). Agricultural landscapes with organic crops support higher pollinator diversity. Oikos.

[47] Privé J.-P., Russell L., LeBlanc A. (2011). Impact of reflective groundcover on growth, flowering, yield and fruit quality in Gala apples in New Brunswick. Can. J. Plant Sci..

