# Supplementary material for: Groundcovers and Rain Shelters Alter Co-Occurrence Patterns among Ground Beetle Communities in an Organic Raspberry Crop
Source: Insects. 2022 Apr 27;13(5):413. doi: 10.3390/insects13050413 (PMC9143038; doi:10.3390/insects13050413)
Supplement: Supplementary file 1 [file insects-13-00413-s001.zip › insects-1661954-Supplementary File S1.pdf]

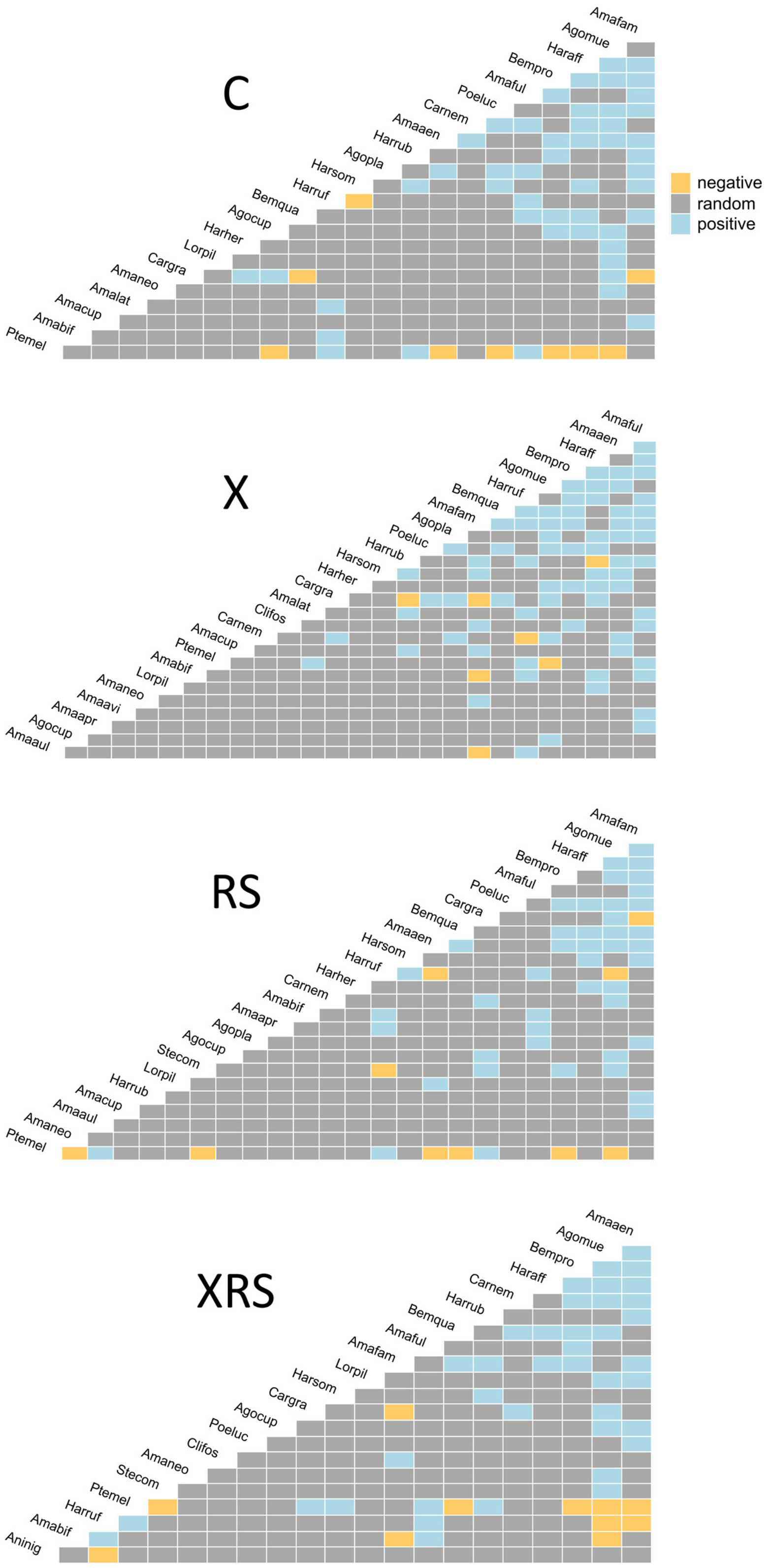

Treatment	Species involved	Positive	Negative	Random	Pair analyzed
C	22	49	9	111	169
X	26	73	7	155	235
RS	24	45	10	132	187
XRS	21	39	11	132	182

Supplementary File S1. Pairwise interactions between species for the four treatments.
