# Supplementary figures and images for: Groundcovers and Rain Shelters Alter Co-Occurrence Patterns among Ground Beetle Communities in an Organic Raspberry Crop
Source: Insects. 2022 Apr 27;13(5):413. doi: 10.3390/insects13050413 (PMC9143038; doi:10.3390/insects13050413)

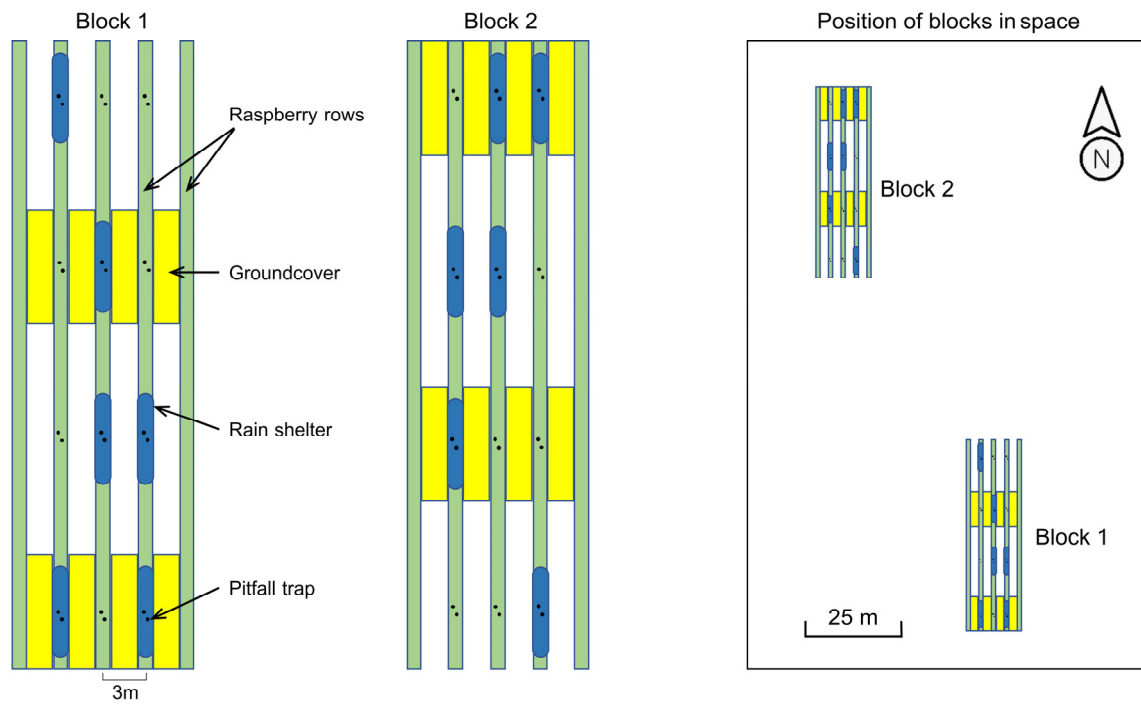

**Figure S1.** Study design.

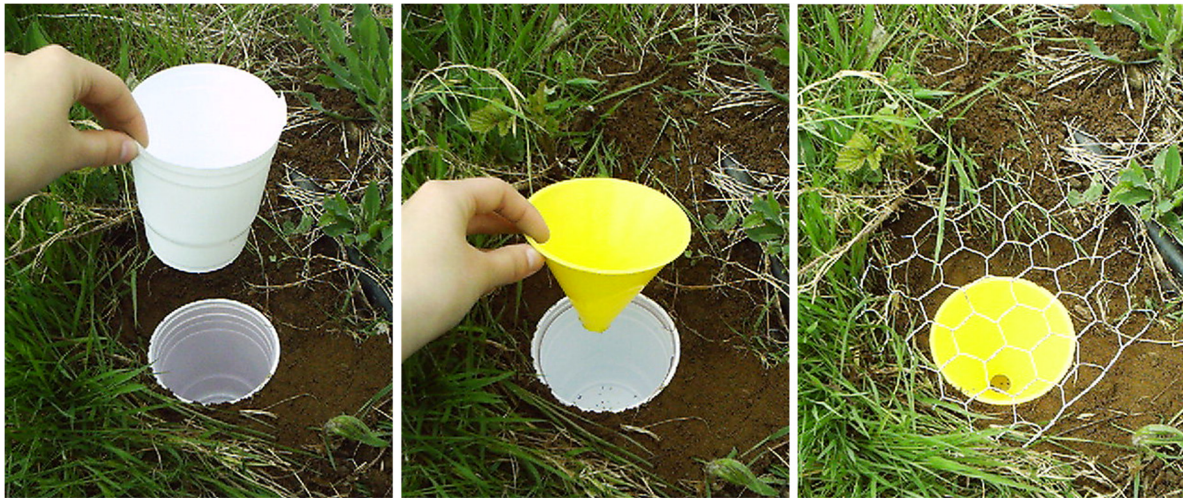

**Figure S2.** Pitfall trap design.

Supplement: Supplementary file 1 [file insects-13-00413-s001.zip › insects-1661954-supplementary.pdf]
